# Structure–Property Relationships of Polymer-Modified Cement Concrete (PCC) Under Service Temperature Conditions

**DOI:** 10.3390/ma18215043

**Published:** 2025-11-05

**Authors:** Alexander Flohr, Savitha Devarajamohalla Narayana, Luise Göbel, Andrea Osburg

**Affiliations:** F. A. Finger-Institute for Building Materials Science, Bauhaus-Universität Weimar, 99423 Weimar, Germanyluise.goebel@uni-weimar.de (L.G.); andrea.osburg@uni-weimar.de (A.O.)

**Keywords:** polymer modification, polymer-modified cement pastes, mortars and concretes, service temperature range, fresh and hardened material properties, hydration kinetics, microstructure, structure–property relationship

## Abstract

Polymer modification is a widely employed technique for optimizing specific properties of mortars and concretes. This process entails the precise tailoring of the binder phase to the requirements of the given application. The polymer addition exerts a significant influence on both the fresh and hardened states of mortar or concrete. In this study, a systematic, stepwise experimental campaign was carried out to investigate the effects of three different polymer dispersions on the time-dependent properties of cement pastes, mortars, and concretes at different temperatures in the service temperature range. The experimental findings demonstrate that polymer modifications significantly influence the behavior of hardened cement-based materials. In general, the strength and deformation resistance decreased with increasing temperature, with this effect being more pronounced in polymer-modified materials. This is indicative of the intrinsic temperature-dependent behavior of the polymers. Temperatures of −20 °C induced specific alterations in the mechanical behavior, particularly evident in the flexural strength and in the early age stiffness development of the pastes, mortars, and concretes. This phenomenon is attributed to the freezing of pore water, which results in the stiffening of the binder structure. In summary, the findings offer significant insights into the structure–property relationships of polymer-modified cement-based materials in relation to temperature.

## 1. Introduction

Polymer-modified mortars and concretes (PCC) have become essential for concrete maintenance, refurbishment, and repair due to their superior workability, chemical resistance, durability, and adhesion compared with unmodified cementitious materials [1,2,3,4]. However, the unique deformation behavior of PCC limits its structural applications, as shown in previous studies addressing the increased elasticity and the pronounced viscoelastic properties of PCC [5,6,7]. Nevertheless, it is important to meticulously investigate and comprehend the constructive behavior of PCC to accurately calculate and predict potential constraining forces and stresses. This is particularly relevant for large-scale repairs of damaged concrete surfaces because it allows us to design materials that can withstand the given constraining stresses without cracking. This maintains the protective effect of the repair layer [8]. Furthermore, this enables the use and adaption of the material properties for innovative applications that go beyond repair and maintenance. These could be, for example, structural applications in which the high durability or specific deformation properties of PCC are advantageous [9]. In addition to the impact of polymers on the properties of mortars and concretes, such as enhanced bonding strength and deformability, it is imperative to consider the temperature-dependent characteristics of the polymers themselves [10]. This inherent property of polymers is likely to exert influence on the characteristics of PCC when exposed to temperatures during their service life. However, the temperature-dependent mechanical properties of such materials have received rare investigation, particularly within the service temperature range. As demonstrated by Konietzko [11], the load-bearing behavior of PCC in the temperature range from −40 °C to 110 °C is predominantly influenced by the thermomechanical properties of the polymers. In the range below the glass transition temperature, the polymer matrix stiffens the binder structure effectively. As the test temperature increases, the binder softens due to alterations in the polymer state. This results in a reduction in strength and modulus of elasticity, accompanied by increased deformability. Kim et al. [12] conducted a study to evaluate the temperature-dependent degradation in the mechanical properties of ethylene-vinyl-acetate-modified cement mortar. The compressive strength and elastic modulus of the mortar underwent a substantial decline with an increase in temperature up to 400 °C. The influence of temperatures below 20 °C on mortar properties was not part of this study. An investigation was conducted by Göbel et al. [13] to ascertain the impact of different polymer types and dosages on the temperature-dependent viscoelastic characteristics of cement pastes using dynamic-mechanical analysis (DMA). A correlation between the temperature dependency of the viscoelastic features of the polymer-modified cement pastes and the viscoelastic characteristics of the pure polymers has been demonstrated.

The mechanical properties of unmodified mortars and concretes are also known to be affected by temperature. Saemann and Washa [14] were among the first to investigate the variation of mortar and concrete properties with temperature, ranging from approximately 60 °C to 230 °C. Their test results revealed that at temperatures below 20 °C, the strength and modulus of elasticity of mortar and concrete generally increase as the temperature decreases. Despite the fact that the strengths of mortars and concretes were only marginally influenced at the highest temperature compared to 20 °C, a discernible decrease in the modulus of elasticity was observed. In their study, Rostásy et al. [15] studied the alterations of compressive and tensile splitting strength of hardened ordinary Portland cement paste, mortar, and concrete at low temperatures. Accordingly, the compressive and tensile splitting strengths of mortar at −196 °C are contingent upon the moisture content at the time of testing. Oven-dried specimens exhibited an increase of 20% in compressive strength and a decrease of 15% in splitting strength. A substantial increase in strength values was observed, with compressive strength increasing by 80°% and splitting tensile strength increasing by 60%, when the specimens were water-saturated. The strength-enhancing influence of frozen pore water was also demonstrated in [16]. The experimental findings presented in [17] reveal a direct correlation between increasing temperature and the enhanced permeability of cement mortars up to a maximum of 600 °C. The residual compressive strength of the mortars increases up to 160 °C but decreases with a further increase in temperature. Additionally, the elastic modulus exhibits a downward trend as the temperature rises. Thienel [18] found that, up to a temperature of 300 °C, the compressive strength of normal concrete is only marginally reduced initially, whereas the modulus of elasticity decreases by up to 50% at this temperature. Above 300 °C, there is a substantial increase in strength loss, accompanied by a decline in the modulus of elasticity to 20% of its value at 20 °C. In addition to elastic deformations, viscous and plastic deformations are also influenced by temperature. Binder et al. [19] investigated the behavior of thermally activated viscoelasticity of cement paste at temperatures of 20 °C, 30 °C, and 45 °C, and their findings confirmed the temperature dependence of hardened cement paste properties. It has been demonstrated that both the elastic modulus and the creep modulus decrease when the temperature is elevated. In [20], creep tests at 20 °C and 40 °C are compared. The findings indicate that the creep-deformations exhibit an increase in response to higher temperatures, even when the temperature differences are relatively modest.

A paucity of systematic analysis exists with respect to the behavior of the PCC, particularly at sub-zero service temperatures and the influence of temperature on the structure formation and, consequently, the development of hardened material properties. The present study addresses the aforementioned lacuna in order to contribute a more complete understanding of the subject. This study constitutes an experimental cross-scale evaluation of the properties of various modified hardened cement pastes, mortars, and concretes over a defined temperature range. The objective of this evaluation is twofold. First, we aim to assess the influence of temperature on the hardening process and on the load-bearing and deformation behavior of PCC, and second, we aim to identify the relationships between microstructure formation and macroscopic material behavior. Accordingly, the degree of hydration, the structure and strength development, and the moduli of elasticity were characterized while the samples were exposed to service temperatures of −20 °C, 20 °C, and 60 °C, which correspond to the maximum, minimum, and middle range of the service temperature range of a Central European climate. The evolution of the microstructure, which is temperature-dependent, could thus be directly correlated to the macroscopic material properties, which represents a further novelty of the present study.

## 2. Materials and Methods

The experimental program was conducted in consecutive steps under varying thermal conditions. The materials were prepared and mixed at a temperature of 20 °C. In the initial phase of the study, the properties of fresh paste, mortar, and concrete were examined, including consistency, air void content, and fresh bulk density. Subsequently, an investigation was conducted into the hardened-state properties of the pastes, mortars, and concretes (flexural strength, compressive strength, static modulus, and dynamic modulus of elasticity) at temperatures of −20 °C, 20 °C, and 60 °C. The reaction processes, microstructure formation, and pore space development were characterized for the given temperatures. This step-by-step systematic approach allows us to identify specific structure-property relationships, analyze the temperature-dependence of paste, mortar, and concrete properties, and discover correlations.

### 2.1. Materials

All experiments were conducted using an ordinary Portland cement (CEM I 42 5 R) provided by Opterra GmbH (Karsdorf, Germany). The cement exhibited a density of 3.11 g/cm^3^, an average particle size of 14.29 µm, and a specific surface (Blaine) of 3580 cm^2^/g. The chemical composition, determined by means of inductively coupled plasma optical emission spectrometry (ICP-OES), is summarized in Table 1.

The clinker phases shown in Table 2 were calculated based on the chemical composition using the Bogue equation [21]. Reported values represent theoretical estimates; deviations from 100% arise from gypsum/anhydrite, free lime, and minor phases not captured by the Bogue approach.

The modification was achieved through the incorporation of three distinct thermoplastic polymer dispersions: styrene-acrylate copolymer (SA), ethylene-vinyl acetate copolymer (EVA), and styrene butadiene rubber copolymer (SBR). All three polymer dispersions contained water as the liquid phase and are commercially available. They are specifically suitable for application in cementitious materials, meaning that they are saponification-stable in an alkaline environment. The material characteristics of the dispersions are summarized in Table 3. The polymers demonstrate divergent temperature-related properties, characterized by their glass transition temperatures (T_g_), minimum film formation temperatures (MFT), and melting temperatures (T_m_). The viscoelastic behavior of the modified cement pastes is governed by T_g_, which is the decisive factor in this system [22]. In accordance with the established processing and storage conditions of 20 °C and 65% rel. humidity, the EVA and SBR polymers form continuous films in hardened cementitious material. It has already previously been shown that SA rarely produces films under these conditions [23]. The polymer particles do not merge, but they form an adhesive bond with aggregates and the hardened cement matrix [4,24,25]. It is well known that in the immediate aftermath of the mixing process, a subset of the polymer particles adsorbs on the surface of the cement clinker grains. The residual part remains dispersed in the pore solution. The polymer particles compelled to coalesce as a result of the continuous growth of CSH phases during the hydration process. During this chemical reaction, a portion of the mixing water is consumed, while another portion evaporates. Consequently, particles of the polymers with MFTs below the production and storage temperatures form films (EVA and SBR), and polymers with higher MFTs at least form an adhesive bond with the remaining structural components (SA) [26].

The cement pastes were prepared using a laboratory mixer from TESTING Bluhm & Feuerherdt GmbH (Berlin, Germany). The initial step involved the mixing of cement and water at a speed of 140 revolutions per minute (rpm) of 30 s. This was followed by adding of the respective polymer dispersion, which was then subjected to a mixing process at 285 rpm for an additional 30 s. Subsequently, the mixing process was paused for a period 90 s. Within this time, all paste adhered to the inner wall and bottom of the mixing vessel, which was then removed using a plastic scraper and added to the center of the vessel. The mixing process was then continued for 60 s at 285 rpm. The total water-to-cement (*w*/*c*) ratio was 0.4. The polymer-to-cement (p/c) ratios were set at 0.05 and 0.15 to elucidate the impact of varying polymer contents on the properties of the cement pastes. The p/c ratio only considers the solid polymer content of the dispersions (Table 3), related to the weight of the cement. The water content of the dispersions was taken into account in the amount of water added. The composition of the cement pastes is presented in Table 4. The addition of polymers to cementitious materials changes the evolution of the pore structure, resulting in altered pore size distributions. According to Zhang [27] and Ramli [28], this alteration depends on the surface charge of the polymers.

To ensure comparability, the mortars were produced with an identical water-to-cement ratio of 0.4 and with the same polymer-to-cement ratios of 0.05 and 0.15. In accordance with DIN EN 196-1 [29], the aggregate-to-cement ratio (a/c) was specified at 3.0 using naturally rounded quartz sand with a maximum grain size of 2 mm and a density of 2.65 g/cm^3^. The composition of the mortars is delineated in Table 5. The mixing processes were executed in accordance with DIN EN 196-1, employing a laboratory mixer from TESTING Bluhm & Feuerherdt GmbH with an automatic preset mixing program that proceeded as follows. Water and cement were mixed at 140 rpm for a duration of 30 s. Subsequently, the sand was added uniformly over a period of 30 s. After that, the mixing process was continued at 285 rpm for a duration of 30 s. Then, the mixer was paused for 90 s, and all mortar that adhered to the inner wall and bottom of the mixing vessel was removed using a plastic scraper and added to the center of the vessel. The mixing process was then continued for 60 s at 285 rpm.

The production of the concretes involved the utilization of the materials and compositions analogous to that employed in the fabrication of pastes and mortars. These materials were complemented by naturally rounded quartzite coarse aggregates, exhibiting grain sizes ranging from 2 mm to 16 mm with a density of 2.60 g/cm^3^. The polymer-to-cement ratios were 0.05 and 0.15, and the total water-to-cement ratio was 0.4. The sand and coarse aggregates were blended to produce a mixture in the middle range of grading curve AB 16 according to DIN 1045-2 [30]. The cement content was set at 340 kg/m^3^, and the amount of the other components was calculated for one cubic meter from the feedstock volumes. The polymer-modified concretes were produced and processed in a manner consistent with the reference concrete. However, it must be noted that the addition of polymers adds a material component to the fresh concrete. This must be taken into account in the material space calculation, which results in one cubic meter of polymer-modified concrete in Equation (1) [9].

(1) $$1\;[m^{3}]=\frac{C}{\rho_{c}}+\frac{W}{\rho_{w}}+\frac{A}{\rho_{a}}+\frac{P}{\rho_{p}}+AV$$

with:

C, W, A, P: added quantity [kg] of the components, cement C, water W, aggregate A, Polymer P
AV: air void content [m^3^]
ρ_c,w,a,p_: bulk densities of material components C, W, A, and P [kg/m^3^]

The following mixing regime was defined to produce the concrete using the laboratory mixer Zyklos ZZ 75 HE from Pemat Mischtechnik GmbH (Freisbach, Germany). The sand and coarse aggregates were first mixed for 30 s. Subsequently, cement was added, followed by a further 60 s of mixing with water and, if necessary, the respective polymer dispersion being added after the 15th to 30th second. After a pausing period of one minute, the mixture was subjected to a secondary mixing process of 30 s. The composition of the concretes is given in Table 6.

The sample designations are as follows. The initial letter column serves to categorize the substance as a paste (PCP), mortar (PCM), or concrete (PCC). The subsequent column delineates the type of modification, with ‘Ref’ signifying an unmodified reference, ‘SA’ denoting styrene acrylate, ‘EVA’ representing ethylene vinyl acetate, and ‘SBR’ indicating styrene butadiene. The following numerical values delineate the percentage polymer content relative to the cement content.

After the production of pastes, mortars, or concretes, along with the completion of the fresh material tests, the materials were poured into special molds to produce the samples necessary for the various tests. The specimens were stored in the molds for a period of 20 h at 20 °C. They were covered with plastic sheets and then demolded. If the specimens were not analyzed immediately or transferred to the appropriate service temperature of −20 °C, 20 °C, or 60 °C, they were stored for a period of 6 days underwater to support the hydration reaction of the cement. Then, the samples were stored at a temperature of 20 °C and a relative humidity of 65%, which favored the formation of polymer films through internal curing until the respective test date. This procedure supports the formation of both binder systems, organic and inorganic [31].

### 2.2. Methods

#### 2.2.1. Properties of the Fresh Materials and Their Hydration Kinetics

The fresh mortar and concrete properties were characterized by determining the consistency using the slump test or spread flow in accordance with DIN EN 1015-3 [32] and DIN EN 12350-5 [33]. The air void content was tested by means of the pressure equalization method with the air void pot in accordance with DIN EN 1015-7 [34] and DIN EN 12350-7 [35], whereby 1 L of mortar or 8 L of concrete were analyzed. The fresh bulk density was identified through the measurement of volume and weight in accordance with DIN EN 1015-6 [36] and DIN EN 12350-6 [37].

The heat rate and cumulative heat release of the cement pastes with and without modifying polymers were studied with at least two samples of the cement pastes using differential scanning calorimetry (DSC) at 20 °C and 60 °C over a period of 168 h using the ToniCal Trio 2 isothermal differential calorimeter (Berlin, Germany), which was first calibrated at the appropriate measurement temperature. The individual components were tempered in the device, then the liquid phase was added to the cement. The measurement was initiated subsequently.

Additionally, the loss on ignition (LOI) at predetermined testing times (1 d, 2 d, 7 d, and 28 d after production) was measured following storage of the samples at temperatures of −20 °C, 20 °C, and 60 °C. Consequently, determination of the chemically bound water was possible. The degree of hydration (DOH) was subsequently calculated as the ratio of the cumulative heat release to the maximum possible heat release (Equation (2)) or as the ratio of the non-evaporable, chemically bound water to the maximum water content that can be bound by the cement used, taking into account its specific composition (Equation (4)).

(2) $$DOH=\frac{Q_{t}}{Q_{max}}\cdot 100$$

with:

DOH: degree of hydration [%]
Q_t_: heat release at a certain time [J/g]
Q_max_: maximum possible heat release [J/g]

The maximum possible heat release (Q_max_) was calculated (Equation (3)) considering the composition of clinker phases of the cement used [38] and their single heat release potential [39].

(3) $$Q_{max}=x_{C3S}\cdot Q_{C3S}+x_{C2S}\cdot Q_{C2S}+x_{C3A}\cdot Q_{C3A}+x_{C4AF}\cdot Q_{C4AF}$$

with:

x_i_: proportion of the clinker phase i [g/g]
Q_i_: single heat release potential of the clinker phase i [J/g]

(4) $$DOH=\frac{m_{w,chem}}{m_{w,max}}\cdot 100=\frac{m_{i}-m_{0}-\frac{{LOI}_{pol}\cdot \frac{p}{c}\cdot m_{0}}{1+\frac{p}{c}-\frac{p}{c}\cdot {LOI}_{pol}}}{m_{w,max}}\cdot 100$$

with:

m_w,chem_: chemical bounded water content at a certain time [g]
m_w,max_: maximum water content that can be bound [g]
m_i_: initial weight of the sample [g]
m_0_: output weight of the ignited sample [g]
LOI_pol_: loss on ignition of the polymer [g/g]
$\frac{p}{c}$: polymer–cement ratio [g/g]

The maximum water content that could be bound was calculated (Equation (5)) considering the composition of clinker phases of the cement used [38] and their single water bound potential [40].

(5) $$m_{w,max}=x_{C3S}\cdot m_{w,C3S}+x_{C2S}\cdot m_{w,C2S}+x_{C3A}\cdot m_{w,C3A}+x_{C4AF}\cdot m_{w,C4AF}$$

with:

m_w,max_: maximum water content that can be bound [g]
x_i_: proportion of the clinker phase i [g/g]
m_w,i_: single water bound potential of the clinker phase I [g]

#### 2.2.2. Temperature-Dependent Microstructural Properties of the Hardened Materials

The microstructural characteristics of the hardened cement pastes were studied, with particular attention paid to the formation of the hydrate phases, the distribution and shape of the unhydrated clinker grains, and the pores. This investigation utilized scanning electron microscopy (SEM) on polished sections of the samples under high vacuum. The Helios Nanolab G4 UX from Thermo Fisher Scientific Inc. (Waltham, MA, USA). was used for this purpose. Even with careful handling, the potential for influences from sample extraction and preparation, as well as from the application of high vacuum, remains unavoidable to a certain extent. In addition, a nanofocus computed tomography system (CT nanotom m “research edition” from General Electric (Boston, MA, USA) was used to obtain a more profound understanding of the three-dimensional distribution of the pores. Software processing was used to generate a three-dimensional model of the samples from the collected data. This model was also employed to gain insights into the pore volume distribution by means of software-supported analyses using VGStudio MAX (Version 2024.1) from Volume Graphics GmbH (Heidelberg, Germany). Mercury high-pressure porosimetry was undertaken to characterize the pore structure analysis in terms of quantitative pore size distribution. To this end, small samples of the pastes measuring approximately 1 cm^3^ were obtained and examined using the AutoPore IV 9500 V1.06 from Micromeritics (Norcrass, GA, USA) at sample ages of 1, 2, and 7 days. The device used is capable of detecting pore radii ranging from approximately 5 nm to 220 µm, with maximum pressures of 206 MPa. The microstructural investigations were conducted at predetermined observation times (2 d, 7 d, and 28 d) following the storage of the samples at temperatures of −20 °C, 20 °C, and 60 °C.

A comprehensive characterization of the hardened pastes, mortars, and concretes was conducted. The bulk and true density were ascertained in accordance with DIN EN 1015-10 [41] and DIN EN 12390-7 [42]. Additionally, the total porosity was calculated by determining the ratio of the dry bulk density to the true density (Equation (6)) of specimens aged for 2, 7, and 28 days.

(6) $$P=1-\frac{\rho_{s,dry}}{\rho }\cdot 100$$

with:

P: total porosity [%]
ρ_s,dry_: dry bulk density [g/cm^3^]
ρ: true density [g/cm^3^]

#### 2.2.3. Temperature-Dependent Mechanical Properties of the Hardened Materials

In accordance with DIN EN 1015-11 [43], DIN EN 12390-3 [44], DIN EN 12390-5 [45], and DIN EN 12390-13 [46], the flexural and compressive strength and the static and dynamic modulus of elasticity of hardened paste, mortar, and concrete specimens were investigated at testing times of 2, 7, and 28 days at temperatures of −20 °C, 20 °C, and 60 °C. The samples were initially stored in the molds for 20 h at 20 °C, covered with foil. Then, the samples were stripped and stored underwater at 20 °C for 6 days, followed by storage at 20 °C and 65% relative humidity until the respective test date. One hour before testing, the samples were thermally conditioned to test temperatures of −20 °C, 20 °C, or 60 °C. Accordingly, the 2 and 7-day-old samples were removed from the water bath, and the 28 d old samples were taken from the climate storage at 20 °C and 65% relative humidity. Then, they were conditioned to the test temperature and finally tested at that temperature.

**Figure 1:**
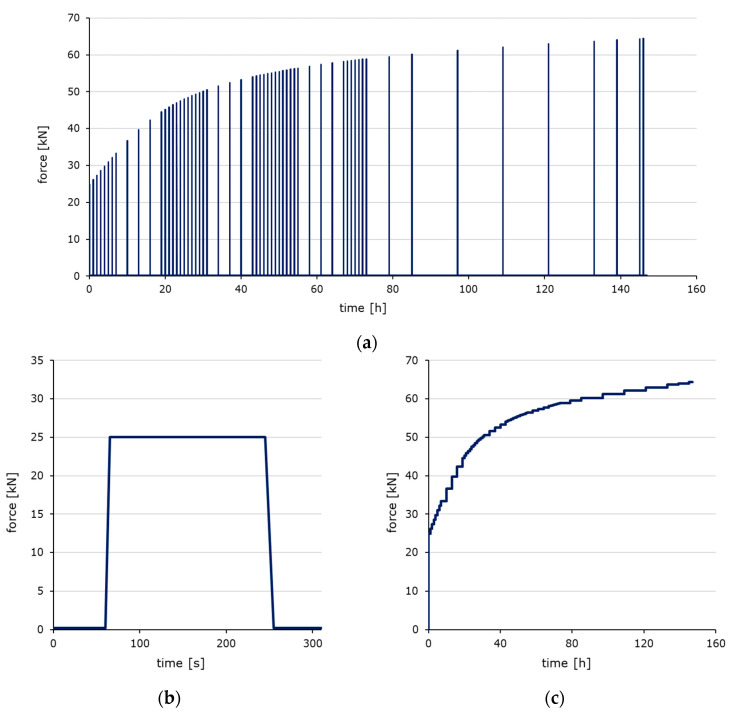
(**a**) Loading regime, (**b**) loading regime of a single load cycle, and (**c**) load plateau values during the testing time.

To determine the stiffness evolution dependent on the temperature, ultrashort loading tests were carried out on hardened pastes and concretes at constant temperatures of −20 °C, 20 °C, and 60 °C. the tests were performed using the universal testing machine TIRAtest 28600 from TIRA GmbH (Schalkau, Germany) with a temperature-controlled test chamber ranging from −40 °C to 180 °C to determine the stiffness evolution dependent on the temperature. To ensure that all samples exhibited an appropriate initial strength, the setting times of the pastes were determined using a Vicat needle device from TESTING Bluhm & Feuerherdt GmbH (Berlin, Germany) to measure the start and end of setting. The ultrashort loading tests were based on the test protocol developed by Irfan-ul-Hassan et al. [47]. Cylindrical specimens with a diameter of 10 cm and a height of 30 cm were used because this geometry ensures a uniaxial stress state in the center area of the test specimens. In this area, the longitudinal compressions along the specimen axis were determined over a length of 100 mm using two inductive displacement transducers arranged opposite each other. The specimens were subjected to compressive loads lasting three minutes at regular intervals, from 21 h after manufacturing up to a material age of 7 days. Due to limitations in data collection and storage, the number of load cycles had to be restricted. For this reason, the load regime was designed so that, from Monday to Thursday, the load was applied hourly from 6:00 a.m. to 6:00 p.m. and every three hours overnight. From Friday to Sunday, the load cycles were executed every five hours. A total of 60 load cycles were performed on each specimen (Figure 1a). Each cycle had a specific load level (Figure 1b) equal to 15% of the expected compressive strength at that time. The respective sample’s compressive strength evolution was obtained by combining its degree of hydration evolution derived from differential scanning calorimetry between 21 and 168 h and the experimentally determined compressive strength of 21 and 168 h old samples stored under the same conditions as the samples for the ultrashort loading tests. Between the single loading processes, the specimens were exposed to a base load of 0.2 kN. The stress rate was 1.0 N/(mm^2^∙s) for the load process and 0.5 N/(mm^2^∙s) for the unload process. The low load level of 15% of the expected compressive strength was chosen to prevent any damage to the material’s structure and plastic deformations.

The elastic modulus E_exp_ of the hardened pastes and concretes was determined as the quotient of the stress difference Δσ and strain difference Δε (Equation (7)) during the unloading phase of each individual loading cycle of the ultrashort load tests.

(7) $$E_{exp}=\frac{\Delta \sigma }{\Delta \varepsilon }$$

with:

E_exp_: elastic modulus [N/mm^2^]
Δσ: stress difference [N/mm^2^]
Δε: strain difference [mm/mm]

## 3. Results

### 3.1. Properties of the Fresh Materials and Hydration Kinetics

#### 3.1.1. Properties of the Fresh Materials

Table 7 and Table 8 show the results of the fresh mortar and fresh concrete tests. The polymers significantly influence the properties of the fresh materials, confirming existing research results [48,49,50]. In general, polymer modification causes liquefaction, improving workability. This effect is attributed to the finely dispersed spherical polymer particles, which create a ball-bearing effect, as well as to the stabilizing additives in the dispersions. Furthermore, the mortars and concretes modified with styrene butadiene rubber copolymer (SBR) exhibit an increased air void content, a phenomenon often described in related publications, particularly those using polymers without defoamer [2]. Entrapped air voids further contribute to the improved workability. The SA-modified mortar and concrete tend to have an entrapped air content that is approximately 2% lower than that of the reference samples. This is due to the polymers’ strong liquefaction, which in turn promotes the deaeration of the samples during compaction. The EVA-modified mortar and concrete have an entrapped air content that is approximately 1% higher than the reference mortar and concrete. Samples modified with SBR have the highest entrapped air content at around 10%, which is significantly higher than that of the reference mortar or concrete. This is related to the use of the SBR-dispersion in tile adhesives rather than in refurbishment or construction applications.

#### 3.1.2. Hydration Kinetics

**Figure 2:**
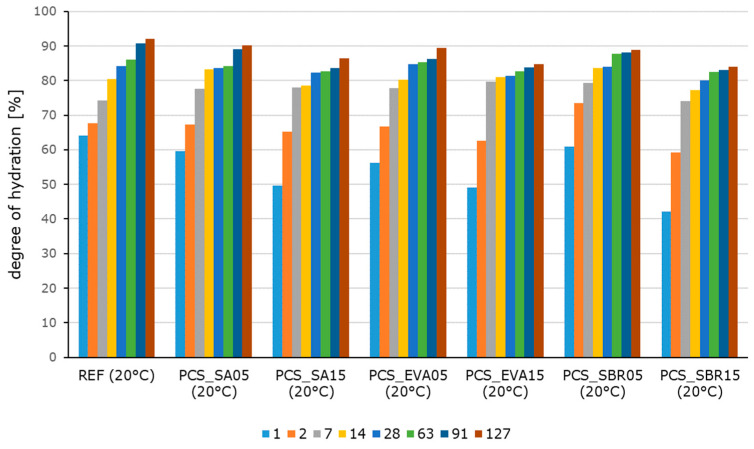
Degrees of hydration of the hardened cement pastes stored at 20 °C after 1 d, 2 d, 7 d, 14 d, 28 d, 63 d, 91 d, and 123 d determined by loss on ignition.

**Figure 3:**
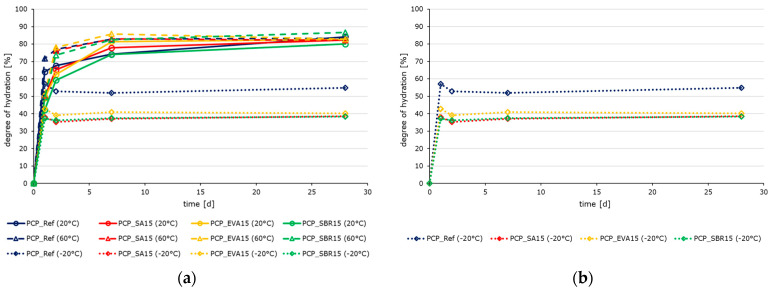

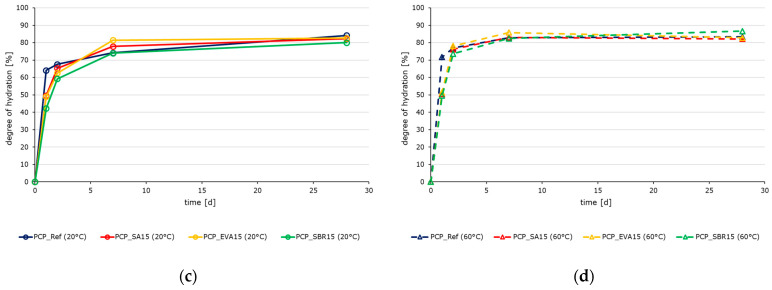
Evolution of the degree of hydration of the reference paste PCP_Ref (blue), the SA-modified paste PCP_SA15 (red), the EVA-modified paste PCP_EVA15 (yellow), and the SBR-modified paste PCP_SBR15 (green) after 24 h, 48 h, 168 h, and 672 h determined by measuring the loss on ignition: (**a**) overview of the cement pastes stored at −20 °C (dotted lines), 20 °C (solid lines), and 60 °C (dashed lines), (**b**) cement pastes stored at −20 °C, (**c**) cement pastes stored at 20 °C, and (**d**) cement pastes stored at 60 °C.

**Figure 4:**
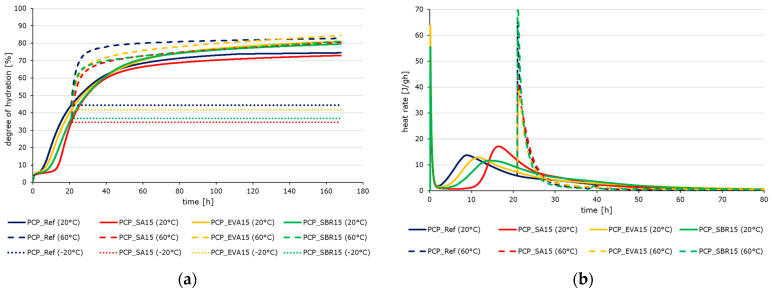
(**a**) Evolution of the degree of hydration of PCP_Ref (blue), PCP_SA15 (red), PCP_EVA15 (yellow), and PCP_SBR15 (green) at −20 °C (dotted lines), 20 °C (solid lines), and 60 °C (dashed lines) until 168 h determined by means of differential scanning calorimetry and (**b**) time-dependent heat rate of the cement pastes stored at 20 °C (solid lines) and 60 °C (dashed lines) until 80 h.

The hydration process of polymer-modified cementitious materials differs from the hydration kinetics of pure cement pastes due to the interaction between the cement and polymer phases. The hydration kinetics of the cement pastes are strongly influenced by the addition of polymers, depending on the polymer content (Figure 2), as well as by ambient temperature (Figure 3 and Figure 4). Contrary to previous reports [51,52,53], polymers generally do not slow the complete hydration process, as indicated by the increased setting times of the pastes (Table 9). As shown in Figure 3 and Figure 4a, the reference paste exhibits a higher degree of hydration than the polymer-modified samples during the first 42 h due to the slowed induction and extended dormant period of the polymer-modified samples (Figure 4b). During the acceleration period, the hydration intensity of the reference paste is lower than that of the polymer-modified samples. This enables the latter to compensate for the slowing almost completely. Initially, temperatures of 60 °C intensify the reaction process at first (see Figure 4b), leading to higher degrees of hydration. This effect is slightly less pronounced in the modified samples. This indicates that the polymers have a slowing effect at higher temperatures due to their post-filming process under these conditions, which prevents unimpeded access of water to the clinker for the hydration. Temperatures of −20 °C cause the hydration reaction to stop, with no further progress detected after the temperature was changed from 20 °C to −20 °C after 21 h. In addition to the thermodynamic aspect, this is also due to the fact that no liquid water is available for hydration, meaning that no ion exchange is possible. The effect of the polymer-modification with a polymer-to-cement ratio of 0.05 is quite marginal. The subsequent investigations were conducted only on the samples with a polymer content of 15% based on the cement content.

### 3.2. Properties and Microstructure of the Hardened Materials

#### 3.2.1. Pore Size Distribution and Microstructure of the Pastes

**Figure 5:**
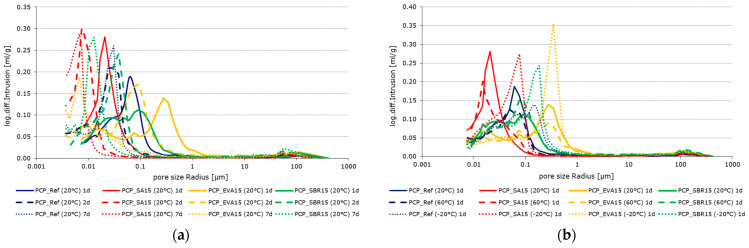
Pore size distribution of PCP_Ref (blue), PCP_SA15 (red), PCP_EVA15 (yellow), and PCP_SBR15 (green): (**a**) stored at 20 °C after 1 d (solid lines), 2 d (dashed lines), and 7 d (dotted lines); and (**b**) pore size distribution of the hardened cement pastes stored at −20 °C (dotted lines), 20 °C (solid lines), and 60 °C (dashed lines) after 1 d.

The pore size distribution of the 1, 2, and 7-day-old cement paste samples stored at 20 °C is shown. As can be seen, the pore size shifts towards smaller radii over the course of one to seven days. This indicates that the content of capillary pores decreases while the content of gel pores increases due to the ongoing hydration process. Figure 5b shows that the temperature applied during the hydration process influences the pore size distribution. Increasing temperatures result in a shift towards smaller radii. As the temperature rises, the hydration rate increases, as does the degree of hydration at the same observation times. Therefore, this shift in pore radius can also be attributed to the progressive hydration.

**Figure 6:**
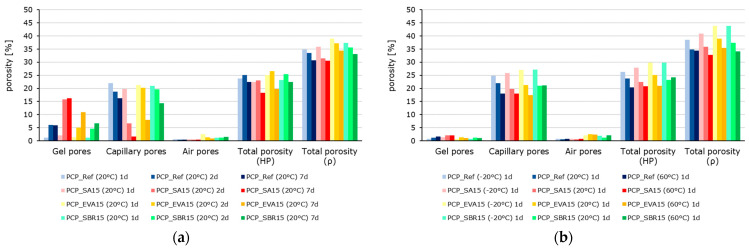
Porosity parameters of PCP_Ref (blue), PCP_SA15 (red), PCP_EVA15 (yellow), and PCP_SBR15 (green): (**a**) time-dependent (the color intensity increases from pale to very dark as the testing time increases from 1 d to 2 d to 7 d) and (**b**) temperature-dependent (the color intensity increases from pale to very dark as the testing temperature rises from −20 °C to 20 °C to 60 °C).

To enable quantitative comparison of the pore fractions, they were classified according to Romberg [54]. To avoid overlap, the capillary pores were defined as ranging from 0.01 µm to 1 µm and the air voids from 1 µm to 1000 µm. Since not all pore fractions are accessible to the mercury, the total ‘porosity (HG)’ determined using this method differs from the total ‘porosity (ρ)’, which was calculated from the raw and pure densities. Figure 6 provides a graphical comparison of the porosity parameters. There is a simultaneous decrease in capillary pores. The capillary porosity decreases significantly with increasing sample age or sample temperature related to hydration. This effect is more pronounced in the polymer-modified samples. This is accompanied by an increase in gel porosity, though not necessarily to the same extent. Overall, total porosity decreases with increasing sample age or storage temperature, which is primarily due to the decrease in capillary porosity.

**Figure 7:**
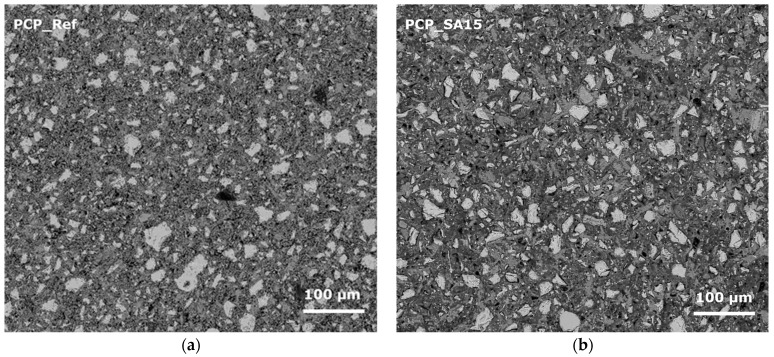

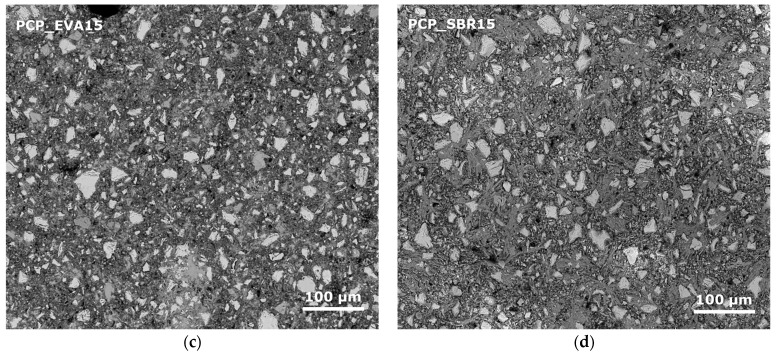
SEM pictures of the microstructure of the hardened cement pastes: (**a**) PCP_Ref, (**b**) PCP_SA15, (**c**) PCP_EVA15, and (**d**) PCP_SBR15 stored at 20 °C after 28 d.

**Figure 8:**
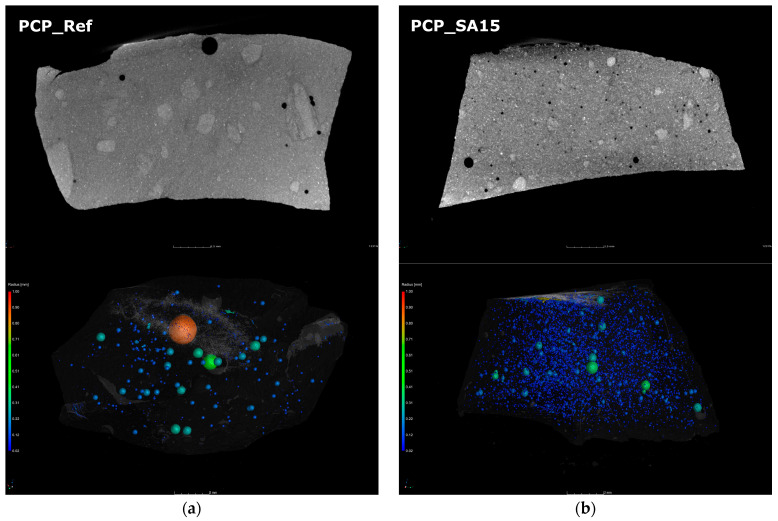

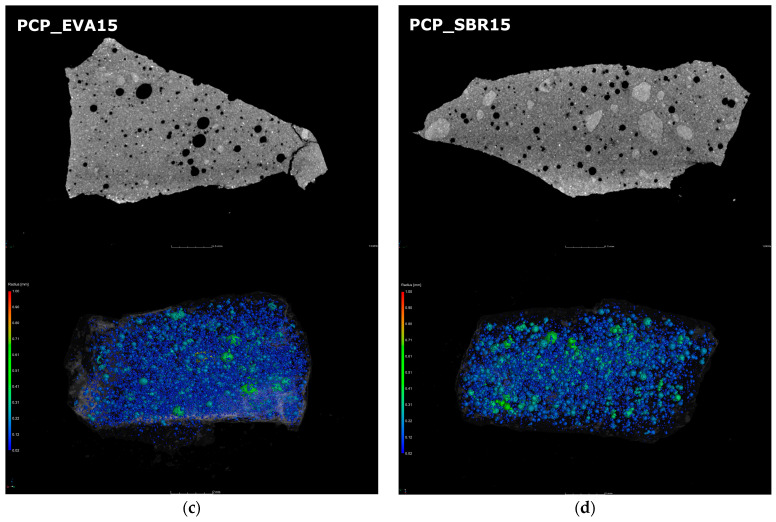
NanoCT images (upper image: 2D section through the sample, lower image 3D reconstruction of the entrapped air voids) of the hardened cement pastes: (**a**) PCP_Ref, (**b**) PCP_SA15, (**c**) PCP_EVA15, and (**d**) PCP_SBR15 stored at 20 °C after 28 d.

**Figure 9:**
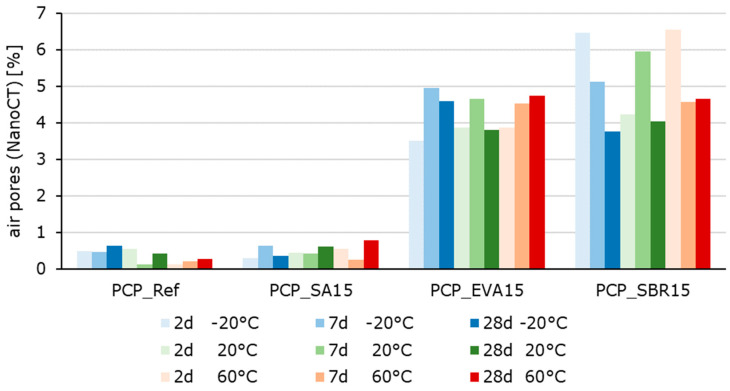
Air void contents of the of the hardened cement pastes determined with nanoCT tested at −20 °C (blue bars), at 20 °C (green bars), and at 60 °C (red bars) from 2 to 7 to 28 days (the color intensity increases from pale to very dark as the sample age increases).

To illustrate the microstructure of the hardened cement pastes at various hydration stages and after storage at temperatures of −20 °C, 20 °C, and 60 °C, scanning electron microscopy (SEM) and computer tomography (CT) in were performed (Figure 7 and Figure 8). The samples were stored at 20 °C for 20 h (protected from evaporation) before being transferred to the respective test temperatures. Thus, it can be assumed that the samples were fully thermally conditioned after 21 h. The samples were then stored under the selected conditions until the time of testing, still covered in foil. The SEM-images show the CSH-phase matrix with embedded compact shaped clinker particles and spherical shaped entrapped air voids. No changes in the distribution, size, or shape of these components were detected as a function of temperature or hydration time. Furthermore, the polymer modification and the varying storage temperatures did not cause any significant differences in the binders’ basic structure. For these reasons, only images of the samples that were 28 days old and stored at 20 °C are shown here as examples (Figure 7). All analyzed samples show the same structural characteristics typical of cement paste. Therefore, when interpreting changes in the properties of solid materials due to polymer modification or different storage conditions, the air voids introduced must be considered especially. Otherwise, the changes in properties are primarily due to the polymers in the structure or to the influence of storage and testing temperatures. Changes in the proportion of unhydrated clinker and the degree of hydration depending on sample age, storage temperature, and formation of CSH-phases cannot be determined from the SEM images. Due to the sample size, it cannot be assumed that the potentially detectable differences are representative of the entire respective material volume, even though the images, which have dimensions of approximately 600 µm to 500 µm, are considerably large for SEM images. The higher air void content of the EVA-modified and SBR-modified cement pastes is evident in CT images (Figure 8) and quantified in Figure 9. This effect was less clearly reflected by mercury intrusion porosimetry or bulk porosity calculations. As with the SEM images, no changes dependent on the degree of hydration or temperature could be detected in the CT images. Therefore, only images of the 28-day-old samples stored at 20 °C are shown here as examples (Figure 8). More detailed information about the microstructure can be found in [13].

The nanoCT analysis (Figure 8 and Figure 9) confirmed a higher content of spherical air voids in the EVA- and SBR-modified systems, which directly corresponds to the hardened material properties shown in in the following sections. These voids originate from air entrapment during mixing, highlighting the combined role of polymer morphology and pore topology in governing the behavior of the hardened materials.

#### 3.2.2. Mechanical Behavior: Temperature-Dependent Flexural and Compressive Strength, Static and Dynamic Elastic Modulus of Polymer-Modified Cementitious Materials

**Figure 10:**
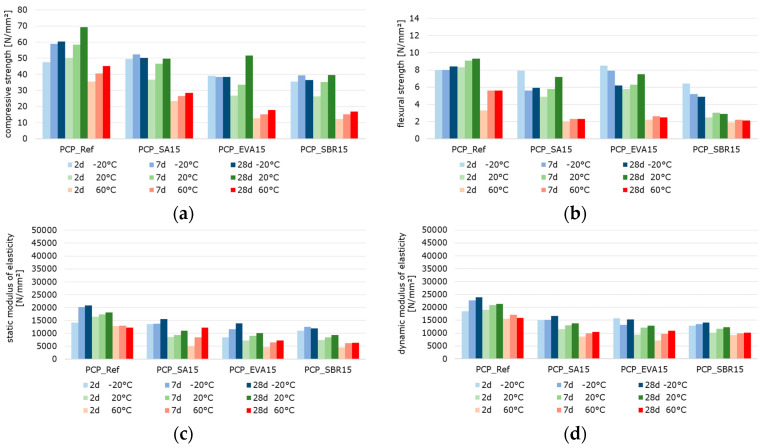
2 d, 7 d, and 28 d strength characteristics of the hardened cement paste samples at testing temperatures of −20 °C (blue bars), 20 °C (green bars), and 60 °C (red bars) (the color intensity increases from pale to very dark as the sample age increases): (**a**) compressive strength, (**b**) flexural strength, (**c**) static modulus of elasticity, and (**d**) dynamic modulus of elasticity.

**Figure 11:**
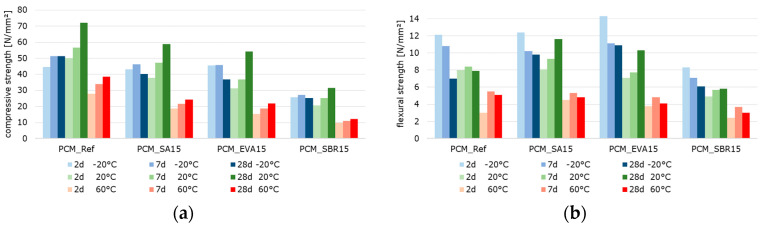

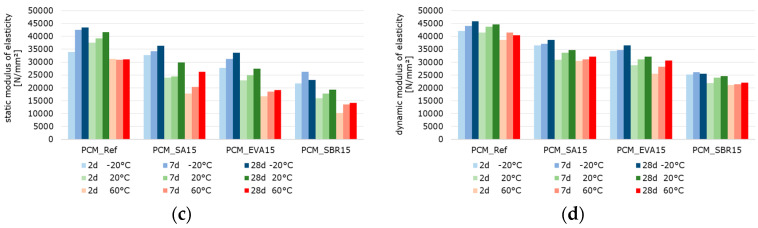
2 d, 7 d, and 28 d strength characteristics of the hardened mortar samples at testing temperatures of −20 °C (blue bars), 20 °C (green bars), and 60 °C (red bars) (the color intensity increases from pale to very dark as the sample age increases): (**a**) compressive strength, (**b**) flexural strength, (**c**) static modulus of elasticity, and (**d**) dynamic modulus of elasticity.

**Figure 12:**
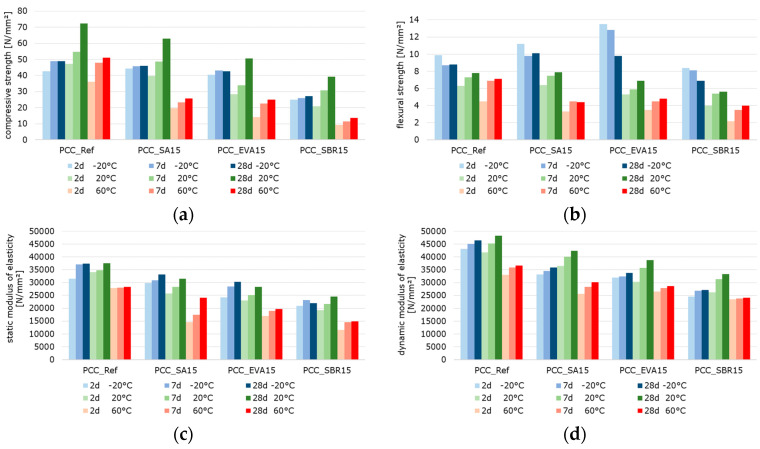
2 d, 7 d, and 28 d strength characteristics of the hardened concrete samples at testing temperatures of −20 °C (blue bars), 20 °C (green bars), and 60 °C (red bars)—(the color intensity increases from pale to very dark as the sample age increases): (**a**) compressive strength, (**b**) flexural strength, (**c**) static modulus of elasticity, and (**d**) dynamic modulus of elasticity.

Figure 10, Figure 11 and Figure 12 summarize the results referring to the temperature-dependent mechanical properties of the hardened cement paste, mortar, and concretes. The air void volume plays a major role in interpreting the results. When analyzing the material properties of the hardened mortar (Figure 11) and concrete (Figure 12), the different air void contents of the fresh mortar and fresh concrete are considered (Table 7 and Table 8) as permanently entrapped air. Also, the air void contents determined with nanoCT (Figure 9) are considered as small spherical voids. These two factors reduce the load-bearing cross-sections of the samples and are thus relevant to the mechanical behavior of the materials.

The results reveal that the flexural strengths, which were determined at −20 °C, decrease with increasing specimen age (Figure 10b, Figure 11b and Figure 12b). This is probably due to the frozen water in the cement paste structure, which contributes to an increase in strength when the samples are young due to its high proportion in the microstructure as well as its strength at low temperatures. In the microstructure of the older samples, the frozen water causes stress due to the increasing strength and stiffness of the cement matrix in combination with the volume expansion of the remaining pore water, resulting in microcracks. These microcracks can transmit forces well under compressive loads but not under tensile loads, such as those occurring in the tensile zone of the bending beams. Thus, they lead to the observed decrease in strength with increasing specimen age.

**Figure 13:**
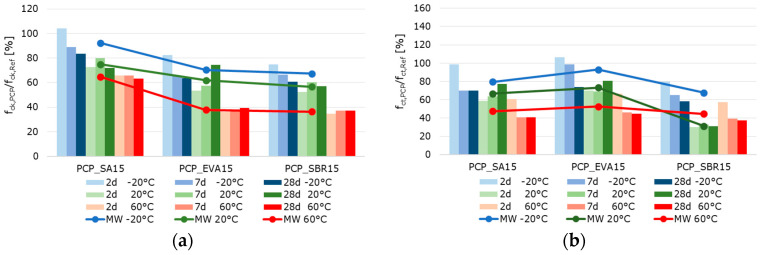

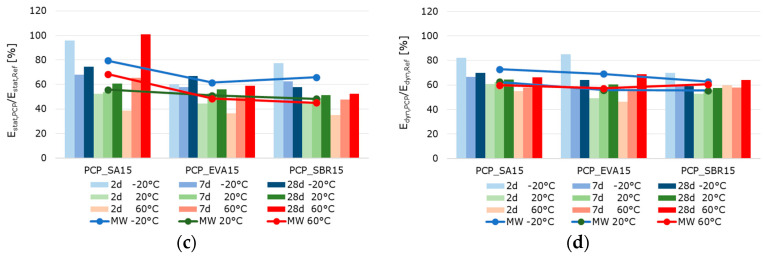
Ratio of the strength characteristics of the hardened polymer-modified pastes to the strength characteristic of the hardened reference paste in percent: (**a**) compressive strength, (**b**) flexural strength, (**c**) static modulus of elasticity, and (**d**) dynamic modulus of elasticity (the blue bars represent the results of the samples tested at −20 °C, the green bars those tested at 20 °C, and the red bars those tested at 60 °C. The color intensity increases from pale to very dark as the sample age increases from 2 to 7 to 28 days).

**Figure 14:**
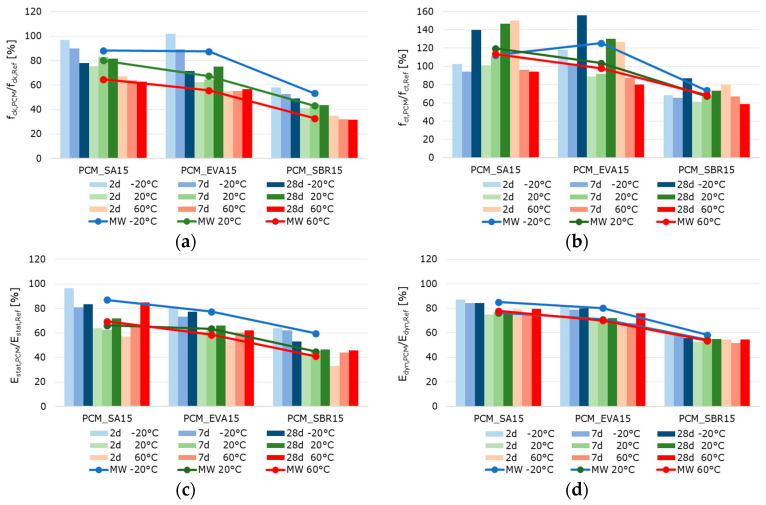
Ratio of the strength characteristics of the hardened polymer-modified mortars to the strength characteristic of the hardened reference mortar in percent: (**a**) compressive strength, (**b**) flexural strength, (**c**) static modulus of elasticity, and (**d**) dynamic modulus of elasticity (the blue bars represent the results of the samples tested at −20 °C, the green bars those tested at 20 °C, and the red bars those tested at 60 °C. The color intensity increases from pale to very dark as the sample age increases from 2 to 7 to 28 days).

**Figure 15:**
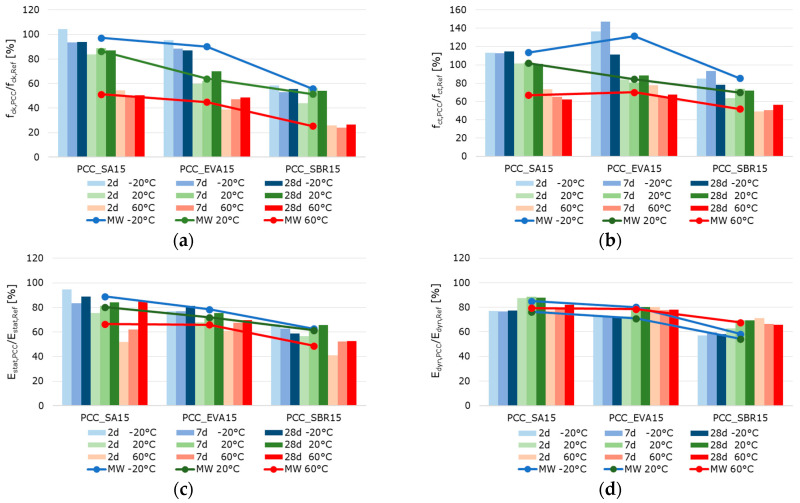
Ratio of the strength characteristics of the hardened polymer-modified concretes to the strength characteristic of the hardened reference concrete in percent: (**a**) compressive strength, (**b**) flexural strength, (**c**) static modulus of elasticity, and (**d**) dynamic modulus of elasticity (the blue bars represent the results of the samples tested at −20 °C, the green bars those tested at 20 °C, and the red bars those tested at 60 °C. The color intensity increases from pale to very dark as the sample age increases from 2 to 7 to 28 days).

The well-documented changes in the properties of hardened cementitious materials due to polymer modification [11,55] are also evident in the test results. In principle, the compressive strength and modulus of elasticity of the polymer-modified materials are lower than those of the unmodified references. This effect is more pronounced at higher temperatures. Polymer-modified cementitious materials reached their maximum strength at temperatures below the polymers’ glass transition temperature. The strength values decrease with increasing temperature, triggered by the polymer softening process. To illustrate the influence of the polymer modification and the different test temperatures, the percentage change in the solid material properties of the polymer-modified materials relative to the reference materials was calculated and is displayed graphically in Figure 13, Figure 14 and Figure 15. The bars represent the deviations of the polymer-modified samples compared to the corresponding reference. The dots represent the averaged time-based deviations, which are connected by lines to illustrate the differences between the modified samples. On average, the compressive strength and the moduli of elasticity of the SA-modified samples were about 20% lower. The samples tested at −20 °C had compressive strengths and deformation resistances that were only around 10% lower than the reference samples tested at the same temperature. The samples tested at 20 °C had compressive strengths and deformation resistances that were approximately 20% lower, and the samples tested at 60 °C had values that were over 30% lower compared to the reference samples tested at these temperatures. This effect was even more pronounced for the compressive strength and moduli of elasticity of the EVA-modified samples. On average, a decrease in compressive strength and deformation resistance of more than 30% compared to the reference samples could be observed. This decrease was still moderate at −20 °C, but exceeded 30% at 20 °C and more than 40% at 60 °C. The low glass transition temperature (T_G_) (Table 3) and the low elastic modulus of EVA already at temperatures of 10 °C led to significant decreases in compressive strength and deformation resistance at 20 °C and 60 °C compared to the reference. The decrease in strength and stiffness across the temperature range was further exacerbated by the increased air void content. It can therefore be concluded that the strength properties and deformation resistance of the EVA-modified materials are significantly more dependent on temperature than those of the SA-modified samples. The SBR-modified samples must be considered separately because of the increased entrapped air content of approximately 10%. The entrapped air influences the strength properties in addition to the modifying polymers. Air voids reduce the load transfer area, leading to a reduction in strength. However, this does not directly correlate with the reduced load transfer area because the spherical air voids also influence the stress distribution in the cross-section, the cracking behavior, and the deformation behavior in a complex manner. On average, the modification of paste, mortar, and concrete with SBR decreases the compressive strength and moduli of elasticity by more than 50%. Even at −20 °C, the compressive strength and deformation resistance are only about 60% of the reference values. At 20 °C they are about 50%, and at 60 °C, they are only about 30%. As previously mentioned, the increased air void content of the SBR-modified samples, in addition to the polymers in the microstructure, is responsible for this reduction in strength and stiffness compared to the references.

Typically, the flexural strength of the modified samples is expected to be higher than that of the reference pastes, mortars, or concretes. This effect is only clearly evident in the SA- and EVA-modified mortars, which have flexural strengths approximately 15% and 10% higher than the reference mortar’s, respectively (Figure 14). For concretes modified with SA and EVA, the increase in flexural strength due to the polymer modification is only significantly pronounced at −20 °C. At 20 °C, the flexural strength of the SA-modified concrete remains nearly unchanged, while the EVA-modified concrete’s strength is 15% lower than the reference concrete’s (Figure 15). The flexural strengths of the modified cement pastes and all SBR-modified samples are generally lower than that of the corresponding reference samples. This behavior was to be expected due to the increased entrapped air content of the SBR-modified materials.

**Figure 16:**
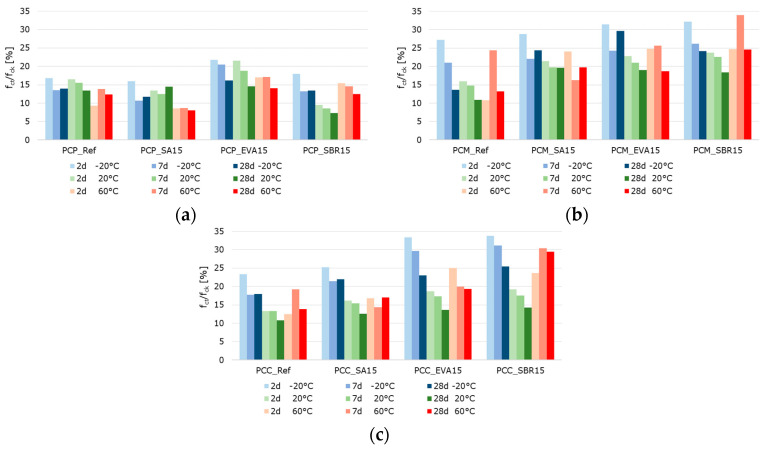
Ratio of flexural strength to the compressive strength of the (**a**) pastes, (**b**) mortars, and (**c**) concretes in percent (the blue bars represent the results of the samples tested at −20 °C, the green bars those tested at 20 °C, and the red bars those tested at 60 °C. The color intensity increases from pale to very dark as the sample age increases from 2 to 7 to 28 days).

The effect of polymer modifications and the associated improvement in the adhesive bond of the structural components of mortars and concretes is evident when examining the flexural-to-compressive strength ratios, particularly in the mortars and concretes (Figure 16). On average, the flexural strength of the reference cement paste corresponds to around 14% of the compressive strength. However, the values are only around 11% for the SA-modified and 12% for the SBR-modified hardened cement paste samples, respectively. Contrary to expectations, no increase in flexural strength due to polymer modification was observed in the hardened SA- or SBR-modified cement pastes, in either the absolute values or the flexural-to-compressive strength ratios. Only the EVA-modified cement pastes showed a higher ratio of flexural-to-compressive strength, with an average of 18%. The influence of polymer modification on the flexural strength of the mortar and concrete samples was more evident. While the flexural strength of the reference mortar and concrete corresponds to around 15% of the compressive strength, the corresponding values are 23% and 18% for the SA-modified mortar and concrete, 24% and 22% for the EVA-modified mortar and concrete, and around 25%for the SBR-modified mortar and concrete. Therefore, it can be concluded that the polymer modifications increase the flexural strength of mortars and concretes, though this does not correlate with the properties of hardened cement pastes in the context of this study. Shrinkage deformations during the hydration process led to stresses in the structure of hardened cement pastes. This was particularly pronounced in the pure cement paste samples. If these stresses exceed the tensile strength of the material, microcracks will occur. As previously mentioned, these microcracks have a significant impact on the flexural strength. Due to the complexity of the influence of polymers on strength development and shrinkage behavior, the exact reason why polymer modifications do not increase the flexural strength of the hardened cement pastes cannot be determined within the scope of this study. However, it can be concluded that the flexural strength of hardened cement paste samples does not reflect the influence of polymer modifications on the flexural strength of mortars and concretes.

When analyzing the temperature dependence of the strength and stiffness properties of the hardened pastes, mortars, and concretes, it was found that, in most cases, a test temperature of −20 °C led to an increase in the compressive and flexural strength, as well as in the moduli of elasticity compared to samples tested at 20 °C. A test temperature of 60 °C significantly reduced the strength and deformation resistance, which is well-documented in literature on the behavior of cementitious materials exposed to elevated temperatures [56,57]. The increase in strength at −20 °C was particularly evident in the flexural strength, while the reduction in strength at 60 °C was evident in the compressive strength. A test temperature of −20 °C caused a slight reduction of approximately 5% in strengths and an increase of 5% in the moduli of elasticity of the reference cement paste compared to the samples tested at 20 °C. A test temperature of 60 °C caused a decrease in strength and deformation resistance. This was most noticeable in the flexural strength of the reference cement paste, which decreases by almost 50%. The SA-modified cement paste exhibited significantly higher strengths and moduli of elasticity at a test temperature of −20 °C than at 20 °C. This effect was most pronounced in the static modulus of elasticity, which increased by almost 50%. At a test temperature of −20 °C, the EVA-modified cement paste exhibited approximately 10% higher compressive strength, 15% higher flexural strength, and 30% higher deformation resistance than at 20 °C. Testing at 60 °C significantly reduced strength by around 60%. The static modulus of elasticity of the EVA-modified cement paste was approximately 30% lower, while the dynamic modulus of elasticity was only around 20% lower at 60 °C. The SBR-modified cement paste showed a similar behavior, with approximately 10% higher compressive strength, nearly 100% higher flexural strength, over 40% higher static modulus of elasticity, and around 20% higher dynamic modulus of elasticity for samples tested at −20 °C compared to the values determined at 20 °C. The SBR-modified pastes demonstrated a substantial decline in strength and stiffness when tested at 60 °C. This decrease was particularly pronounced with more than 50% reduction in compressive strength, around 25% in flexural strength, over 30% in the static modulus of elasticity, and approx. 15% in the dynamic modulus of elasticity compared to the values recorded at 20 °C.

As expected, the mortars and concretes behaved similarly to hardened pastes at varying test temperatures. At a test temperature of −20 °C, the compressive strength of the reference mortar or concrete decreased by more than 15%, while the flexural strength increased by slightly more than 25% compared to the values determined at 20 °C. The moduli of elasticity of the reference mortar and concrete remained nearly unchanged compared to the samples tested at 20 °C. Testing at 60 °C decreased strength and deformation resistance, most notably in the flexural strength of the reference mortar, which decreased by almost 45%. The SA-modified mortars and concretes exhibited significantly higher flexural strengths and static moduli of elasticity at a test temperature of −20 °C than at 20 °C. The EVA-modified mortar and concrete exhibited approximately 15% higher compressive strength, 50% to 100% higher flexural strength, and about 10% higher deformation resistance at a testing temperature of −20 °C compared to the samples tested at 20 °C. Testing at 60 °C significantly reduced strength by around 50%. The moduli of elasticity tested were also lower at 60 °C, with the static modulus of elasticity being approximately 25% lower and the dynamic modulus of elasticity being only around 15% lower. The compressive strengths of the SBR-modified mortar and concrete tested at −20 °C remained almost unchanged, while the flexural strength increased by over 30% for the mortar and over 60% for the concrete. The static modulus of elasticity of the SBR-modified mortar increased by more than 30%, while the static modulus of the concrete remained unchanged. The dynamic modulus of elasticity of the SBR-modified mortar increased by nearly 10%, while the dynamic modulus of elasticity decreased by 15% compared to the values determined at 20 °C. The decrease in the strength and stiffness of the SBR-modified mortars at a testing temperature of 60 °C is significant, over 55% for compressive strength, about 45% for flexural strength, over 30% for the static modulus of elasticity, and approximately 15% for the dynamic modulus of elasticity. In summary, the properties of cementitious materials are, as expected, significantly influenced by polymer modifications and depend on the sample temperature at the time of testing. This influence is more pronounced in the modified materials; that is, the increase in strength deformation resistance at low temperatures, as well as the decrease in strength and deformation resistance at higher temperatures, occur to a greater extent than in the unmodified references. At a testing temperature of −20 °C, the frozen pore water and the glassy state of the polymers temporarily increase the stiffness of the composite, enhancing the apparent stiffness and flexural strength. However, differential shrinkage and ice-induced microcracking counteract this effect at later ages. At 20 °C, hydration and polymer coalescence proceed simultaneously across the different polymer dispersions, resulting in balanced microstructures. At 60 °C, the softening of the polymer phase above the glass transition temperature further reduces the load transfer capability, explaining the steep decline in deformation resistance.

#### 3.2.3. Mechanical Behavior: Hydration-Dependent Development of the Moduli of Elasticity at Different Temperatures

**Figure 17:**
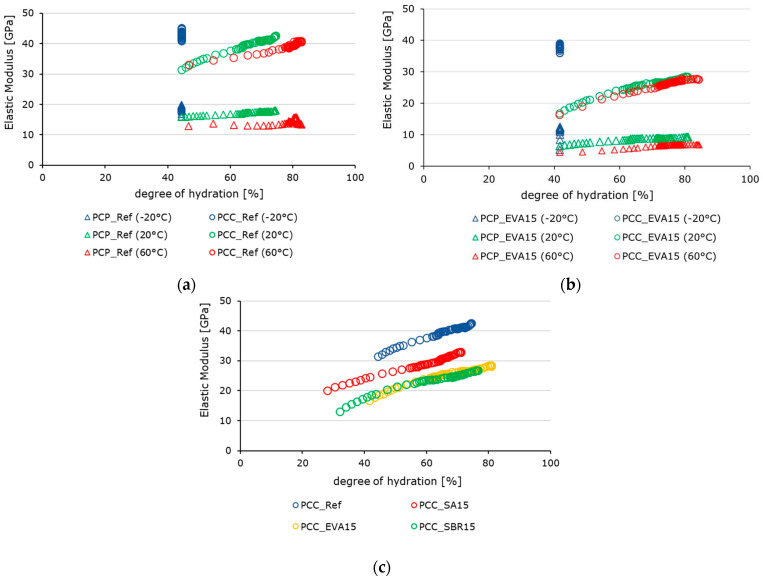
Hydration-dependent development of the moduli of elasticity of (**a**) the reference cement paste (triangles) and the reference concrete (circles) in comparison to (**b**) the EVA-modified cement paste (triangles) and concrete (circles) at −20 °C (blue), 20 °C (green), and 60 °C (red), as well as (**c**) the hydration-dependent development of the moduli of elasticity of the reference concrete and the reference concrete (blue), the SA-modified concrete (red), the EVA-modified concrete (yellow), and the SBR-modified concrete (green) at 20 °C.

Figure 17 shows the hydration-dependent development of the moduli of elasticity of the reference cement paste, the reference concrete, and the EVA-modified cement paste and concrete at different temperatures as well as the hydration-dependent development of the moduli of elasticity of the reference concrete in comparison to the polymer-modified concretes at 20 °C. The values were determined in the ultrashort loading tests. Figure 17a,b illustrate the evolution of the modulus of elasticity (*y*-axis) versus the degree of hydration (*x*-axis) for hardened cement pastes and concretes at the distinct test temperatures of −20 °C, 20 °C, and 60 °C. Figure 17c illustrates the evolution of elastic moduli (*y*-axis) in relation to the degree of hydration (*x*-axis) for the reference concrete, the SA-modified concrete, the EVA-modified concrete, and the SBR-modified concrete.

The evolution of the elastic modulus as a function of the degree of hydration reveals a clear interrelation between microstructural development, temperature, and polymer modification. The elastic moduli of the polymer-modified samples are generally lower than those of the corresponding reference samples (Figure 17). This correlates with the results of the hardened material characterization from the previous section and findings in the literature [58]. This behavior is attributed to the softer polymer phase. While SA induces moderate reductions, EVA and SBR cause the most pronounced decrease in elastic modulus due to their increased air void and entrapped air contents. The curves showing the development of the moduli of elasticity are largely parallel (Figure 17c), meaning that the initially delayed hydration reaction of the polymer-modified materials is not immediately reflected in the evolution of stiffness.

In terms of temperature, the highest stiffness values were observed for the samples tested at −20 °C due to the stiffness-enhancing influence of frozen pore water. Storage at this temperature causes the hydration reaction to cease. Thus, the degree of hydration does not progress. Accordingly, these values correspond to the respective degree of hydration achieved after 21 h and do not change (Figure 17a,b). The lowest deformation resistance was observed in the samples tested at 60 °C. The influence of increasing temperatures on the elastic modulus is less pronounced in the EVA-modified samples than in the reference samples, despite the fact that the stiffness of the modified samples is significantly lower. This result apparently contradicts the values determined in the context of hardened material characterization (Figure 10, Figure 11 and Figure 12). However, at this early stage of hardening, the reduction in stiffness caused by increased temperature is likely to be offset by the accompanying acceleration of the reaction. As described in Section 2.1, the samples for material characterization were stored at 20 °C until the respective test date. The ultra-short-term tests were carried out immediately after demolding the samples at the respective test temperatures of −20 °C, 20 °C, and 60 °C, which naturally affects the hydration process during the load tests.

Finally, it must be taken into account that microcrack-inducing structural stresses cannot be excluded, especially in the tests on the pure cement pastes, as previously mentioned and explained. Additionally, hydration-related heat development influences the reaction kinetics, particularly in the first 48 h. For all samples, moisture release from the samples cannot be completely ruled out, despite the careful use of film to prevent it.

## 4. Conclusions

This study offers novel insights into the impact of service temperature on the structural and physical characteristics of polymer-modified cementitious materials (PCC). A comprehensive examination of these materials was conducted across a range of scales encompassing cement pastes, mortars, and concretes modified with dispersions of copolymers of styrene–acrylate (SA), ethylene–vinyl acetate (EVA), and styrene–butadiene rubber (SBR). These materials were subjected to experimental evaluation within a defined temperature range to assess the impact of varying temperatures on the hardening process as well as on their load-bearing and deformation behavior. The degree of hydration, the structure and strength development, and the moduli of elasticity were characterized for samples that were exposed to service temperatures of −20 °C, 20 °C, and 60 °C. The findings of this research indicate that the polymers as well as the altered hydration process and pore structure, resulting from the polymer modification, are the underlying causes of the observed changes in the mechanical behavior of the polymer-modified pastes, mortars, and concretes in comparison to their respective reference materials. Additionally, the results reveal that the temperature-dependent properties of the modifying polymers themselves affect these alterations depending on the respective testing temperature.

The hardening process of the cement pastes is remarkably influenced by the addition of modifying polymers and the application of different temperatures. The polymers initially delay hydration by lowering reaction intensity during the induction phase and prolonging the dormancy period. The hydration degree deficit of the modified cement pastes is then compensated by a higher reaction intensity during the acceleration period. As a result, the polymer-modified pastes demonstrate equivalent reaction progress to the unmodified paste after approximately 40 h. Higher temperatures have been shown to intensify the hydration reactions. However, the effect on the overall degree of hydration is less pronounced than expected. A thorough examination of the microstructure confirmed that the pore structure undergoes distinct evolution in response to variations in polymer type and temperature. The presence of EVA and SBR has been observed to result in a higher content of air voids and entrapped air, thereby influencing the mechanical behavior of polymer-modified cementitious materials. This phenomenon has been identified as the predominant factor when considering the decline in total porosity with increasing temperature. The application of different temperatures within the defined service temperature range during testing of the mechanical behavior results in significant variations in the mechanical parameters of the pastes, mortars, and concretes. In general, the strength and deformation resistance of the materials decreased with increasing temperature, with this effect being more pronounced in polymer-modified materials. Temperatures of −20 °C induced unanticipated alterations in the mechanical behavior, particularly evident in the flexural strength of the pastes, mortars, and concretes. This is attributable to the freezing of water within the pores. These effects manifested most distinctly during the initial hydration stages, when the water content was comparatively elevated and the maximum flexural strengths were recorded. The low-temperature testing revealed the dual role of frozen pore water, which initially increases strength but can also induce microcracking at later ages. The reduction in stiffness and strength of polymer-modified systems can be directly linked to the formation of a continuous polymer phase during hydration, which partially encapsulates the cement hydrates and softens the rigid hydrate-aggregate network. The polymers in the interfacial transition zone reduce the effective stiffness of the load-bearing framework, especially above the glass transition temperature of the polymer. In contrast to the EVA-modified and SBR-modified systems, the SA-modified systems, in which no continuous polymer films are formed under the given curing conditions, exhibit higher stiffness values despite similar degrees of hydration. The increased air void content observed in the EVA- and SBR-modified materials is responsible for the lower load transfer efficiency.

The ultra-short-term loading tests confirmed that the polymer-modification samples consistently exhibited lower elastic moduli than their unmodified references, which aligns with the results from hardened material testing. The development curves of the elastic moduli ran largely parallel, indicating that the initial hydration delay caused by polymers was not immediately reflected in stiffness evolution. The impact of temperature on the system was significant. The highest stiffness values occurred at −20 °C due to the stiffening effect of frozen pore water. However, the hydration was terminated at this temperature. Consequently, the stiffness values corresponded exclusively to the degree of hydration that had been achieved after 21 h. The lowest stiffness was measured at 60 °C, although this reduction was less pronounced in the polymer-modified samples compared to the reference. This apparent contradiction with hardened material tests is explained by the early testing stage. An increase in temperature led to a decrease in stiffness while concurrently stimulating hydration, thereby counterbalancing the initial effect to a certain extent.

The experimental results demonstrate that the evolution of stiffness and strength in polymer-modified cementitious composites is not governed by hydration alone but by the interplay of three coupled mechanisms: first, the temperature-dependent physical state of the polymer phase and its interaction with hydrates; second, the microstructural refinement or coarsening induced by curing temperature; and third, the efficiency of stress transfer across the polymer–hydrate–aggregate interfaces. At sub-zero temperatures (−20 °C), the partial freezing of pore water leads to a temporary increase in apparent stiffness, while the polymer phase remains in a glassy and rigid state. This combination restricts local deformation and produces higher instantaneous elastic moduli. However, the heterogeneous expansion of ice within the capillary network generates microcracking, which subsequently limits strength development. At ambient temperature (20 °C), hydration of the cement and formation of the polymer phase proceed concurrently. The polymer phase remains semi-elastic, allowing for effective load transfer while maintaining tight interfacial contact between hydrates and aggregates. Consequently, these systems exhibit the most balanced combination of strength and stiffness, reflecting a microstructure with low internal stresses and optimal polymer–hydrate compatibility. At an elevated temperature (60 °C), the polymers transition into a softened, thermoplastic state that cannot sustain mechanical loads effectively. The combination of reduced solid volume fraction and softened polymer matrix causes a pronounced decrease in strength and stiffness, even at comparable hydration degrees. The microstructural observations support these interpretations. SEM and nano-CT analyses revealed that EVA- and SBR-modified systems contain a larger fraction of spherical voids than the reference and SA-modified materials, consistent with their lower strength and deformation resistance.

Overall, the results confirm that the macroscopic mechanical response of polymer-modified concrete arises from the complex balance between thermal transitions of the polymer phase, microstructural evolution of the hydrates, and interfacial bonding efficiency. These findings provide a mechanistic foundation for the temperature-dependent performance of PCC materials and highlight the need to consider both physical polymer behavior and chemical hydration processes in future material models. Furthermore, the results emphasize that service temperature is a critical factor in determining the performance of PCC. The pronounced temperature sensitivity of polymer-modified systems underscores the need to consider polymer type, dosage, and environmental conditions in structural design and practical applications.

## Figures and Tables

**Table 1:** Chemical components of the Portland cement in weight percentage.

Chemical Component	CaO	SiO_2_	Al_2_O_3_	Fe_2_O_3_	SO_3_	MgO	K_2_O	Na_2_O	LOI *
Percentage [%]	64.1	19.5	5.0	2.9	3.2	1.5	1.0	0.2	2.2

* LOI—Loss on Ignition.

**Table 2:** Clinker phases of the Portland cement in weight percentage.

Cement Clinker Phase	C_3_S	C_2_S	C_3_A	C_4_AF
Percentage [%]	63.0	8.4	8.3	8.8

**Table 3:** Material properties of the polymer dispersions.

Polymer		SA	EVA	SBR
Main constituents		styrene, acrylic acid ester	ethylene, vinyl acetate	styrene, butadiene
Solid particle content	[%]	50.6	53.7	50.8
Density	[g/cm^3^]	1.03	1.07	1.02
Particle size range	[µm]	0.04–2.11	0.52–7.08	0.08–0.21
Mean particle size	[µm]	0.15	1.34	0.13
pH value at 20 °C	[-]	8.18	3.24	8.04
Particle charge density	[C/g]	−0.13	−0.21	−15.84
Dynamic viscosity (at 20 °C, measurement range between 1 and 120 U/min)	[mPas]	37–490	45–640	220–940
MFT	[°C]	33	0	12
T_g_	[°C]	22	−6	16
T_m_	[°C]	380	330	130

**Table 4:** Composition of the cement pastes.

Sample Designation	Unit	Cement	Water	Polymer Dispersion
PCP_Ref	[kg/m^3^]	1385.9	554.4	-
PCP_SA05	[kg/m^3^]	1298.6	454.5	128.3
PCP_SA15	[kg/m^3^]	1153.2	288.3	341.8
PCP_EVA05	[kg/m^3^]	1301.6	455.6	121.2
PCP_EVA15	[kg/m^3^]	1160.5	290.1	324.1
PCP_SBR05	[kg/m^3^]	1297.8	454.2	127.7
PCP_SBR15	[kg/m^3^]	1160.5	290.1	342.7

**Table 5:** Composition of the mortars.

Sample Designation	Unit	Cement	Water	Polymer Dispersion	Sand 0/2
PCM_Ref	[kg/m^3^]	539.5	215.8	-	1618.5
PCM_SA05	[kg/m^3^]	526.4	184.3	52.0	1579.3
PCM_SA15	[kg/m^3^]	502.2	125.5	148.9	1506.5
PCM_EVA05	[kg/m^3^]	528.9	185.1	49.2	1586.8
PCM_EVA15	[kg/m^3^]	509.0	127.2	142.2	1527.0
PCM_SBR05	[kg/m^3^]	526.3	184.2	51.8	1578.9
PCM_SBR15	[kg/m^3^]	501.7	125.4	148.1	1505.2

**Table 6:** Composition of the concretes.

Sample Designation	Unit	Cement	Water	Polymer Dispersion	Sand 0/2	Aggregates 2/16
PCC_Ref	[kg/m^3^]	340.0	136.0	-	584.1	1337.2
PCC_SA05	[kg/m^3^]	340.0	119.4	33.6	571.3	1308.0
PCC_SA15	[kg/m^3^]	340.0	86.2	100.8	545.9	1249.6
PCC_EVA05	[kg/m^3^]	340.0	121.3	31.7	572.2	1310.0
PCC_EVA15	[kg/m^3^]	340.0	92.0	95.0	548.5	1255.6
PCC_SBR05	[kg/m^3^]	340.0	119.5	33.5	571.1	1307.4
PCC_SBR15	[kg/m^3^]	340.0	86.6	100.4	545.1	1247.8

**Table 7:** Fresh mortar properties.

Sample ID	Slump [mm]	Consistency	Air Void Content [%]	Fresh Bulk Density [g/cm^3^]
PCM_Ref	102	stiff	4.5	2.30
PCM_SA05	122	stiff	4.3	2.27
PCM_SA15	199	plastic	2.8	2.24
PCM_EVA05	108	stiff	4.9	2.27
PCM_EVA15	143	plastic	5.4	2.20
PCM_SBR05	146	plastic	7.2	2.20
PCM_SBR15	217	soft	10.0	2.09

**Table 8:** Fresh concrete properties.

Sample ID	Slump [mm]	Consistency	Air void Content [%]	Fresh Bulk Density [g/cm^3^]
PCC_Ref	355	stiff	3.0	2.39
PCC_SA05	385	stiff	2.5	2.39
PCC_SA15	620	flowable	1.2	2.35
PCC_EVA05	360	plastic	4.1	2.32
PCC_EVA15	370	plastic	3.8	2.30
PCC_SBR05	460	soft	11.3	2.13
PCC_SBR15	650	very flowable	9.5	2.14

**Table 9:** Start of setting of the pastes.

Sample ID	PCP_Ref	PCP_SA15	PCP_EVA15	PCP_SBR15
Setting time [min]	190	865	495	625

## Data Availability

The original contributions presented in this study are included in the article. Further inquiries can be directed to the corresponding author.
